# DIEN Expert System Version 1 to formulate nursing diagnoses 

**DOI:** 10.15649/cuidarte.3945

**Published:** 2025-02-07

**Authors:** María Margarita Fanning Balarezo, María Rosa Vásquez Pérez, Oscar Efrain Capuñay Uceda, Susan Míriam Oblitas-Guerrero, María Alejandra Juárez Elera

**Affiliations:** 1 Universidad Nacional Pedro Ruiz Gallo. Lambayeque- Perú. mfanning@unprg.edu.pe Universidad Nacional Pedro Ruiz Gallo Universidad Nacional Pedro Ruiz Gallo Lambayeque Perú mfanning@unprg.edu.pe; 2 Universidad Nacional Pedro Ruiz Gallo. Lambayeque- Perú. mvasquez@unprg.edu.pe Universidad Nacional Pedro Ruiz Gallo Universidad Nacional Pedro Ruiz Gallo Lambayeque Perú mvasquez@unprg.edu.pe; 3 Universidad Nacional Pedro Ruiz Gallo. Lambayeque- Perú. ocapunayu@unprg.edu.pe Universidad Nacional Pedro Ruiz Gallo Universidad Nacional Pedro Ruiz Gallo Lambayeque Perú ocapunayu@unprg.edu.pe; 4 Universidad Nacional Pedro Ruiz Gallo. Lambayeque- Perú. oblitasg@uss.edu.pe Universidad Nacional Pedro Ruiz Gallo Universidad Nacional Pedro Ruiz Gallo Lambayeque Perú oblitasg@uss.edu.pe; 5 Universidad Nacional Pedro Ruiz Gallo. Lambayeque- Perú. mjuareze@unprg.edu.pe Universidad Nacional Pedro Ruiz Gallo Universidad Nacional Pedro Ruiz Gallo Lambayeque Perú mjuareze@unprg.edu.pe

**Keywords:** Nursing Education, Competency-Based Education, Nursing Diagnosis, Artificial Intelligence, Standardized Nursing Terminology, Educación en Enfermería, Educación Basada en Competencias, Diagnóstico de Enfermería, Inteligencia Artificial, Terminología Normalizada de Enfermería, Educação em Enfermagem, Educação Baseada em Competências, Diagnóstico de Enfermagem, Inteligência Artificial, Terminologia Padronizada em Enfermagem

## Abstract

**Introduction:**

Students and professionals’ difficulty in formulating nursing diagnoses underlines the need to use tools based on expert systems.

**Objective:**

To develop an expert system to formulate nursing diagnoses and to evaluate their attributes.

**Materials and Methods:**

This technological-descriptive study was conducted in two phases. The first phase was the design and construction of the expert system and the evaluation of its attributes (usability, functionality, reliability, and portability). In the second phase, 68 people participated, including students and nurses. Two questionnaires were applied, one to evaluate usability (validated by exploratory factor analysis, with a Cronbach's alpha reliability of 0.93) and the other to evaluate the remaining attributes (validated with Aiken's V: 0.91; with a Cronbach's alpha reliability of 0.91). The data were processed in Excel, using descriptive statistics.

**Results:**

An expert system was designed using the NANDA International 2021-2023 as its knowledge base. Its interface allows users to input age group, characteristics, and factors, generating diagnostic labels. Most users rated the attributes of usability (79.41%), functionality (82.35%), reliability (77.94%), and portability (86.76%) as "very good."

**Discussion:**

The DIEN Expert System Version 1 develops skills to identify characteristics and related or risk factors. It familiarizes users with the standardized nursing language and strengthens critical thinking to formulate contextualized diagnoses for the person cared for.

**Conclusion:**

The DIEN Version 1 enables the use of standardized nursing diagnostic language, as it demonstrates the scientific and systematized work in care. The favorable opinion of its attributes by most participants predicts its acceptance in training and care settings.

## Resumen

**Introducción:**

La dificultad para formular diagnósticos enfermeros en estudiantes y profesionales subraya la necesidad de utilizar herramientas basadas en sistemas expertos.

**Objetivo:**

Desarrollar un sistema experto para la formulación de diagnósticos enfermeros y evaluar sus atributos.

**Materiales y métodos:**

Estudio tecnológico-descriptivo realizado en dos fases: diseño y construcción del sistema experto y evaluación de sus atributos (usabilidad, funcionalidad, fiabilidad y portabilidad). En la segunda fase participaron 68 personas entre estudiantes y enfermeras. Se aplicaron dos cuestionarios, uno para evaluar la usabilidad (validado por análisis factorial exploratorio, con confiabilidad 0,93 alfa de Cronbach) y el otro para los atributos restantes (validado con V de Aiken: 0,91; con confiabilidad: 0,91 alfa de Cronbach). Los datos fueron procesados en Excel, aplicándose estadística descriptiva.

**Resultados:**

Se diseñó un sistema experto, utilizando como base de conocimiento la NANDA Internacional 2021-2023. Su interfaz permite ingresar grupo etario, características y factores, generando etiquetas diagnósticas. La mayoría de usuarios calificó como muy buenos los atributos de usabilidad (79,41%), funcionalidad (82,35%), fiabilidad (77,94%) y portabilidad (86,76%).

**Discusión:**

El sistema experto DIEN Versión 1 desarrolla habilidades para identificar características y factores relacionados o de riesgo, familiariza a los usuarios con el lenguaje estandarizado de enfermería y fortalece el pensamiento crítico para formular diagnósticos contextualizados con la persona cuidada.

**Conclusiones:**

El DIEN Versión 1 facilita el uso del lenguaje estandarizado del diagnóstico enfermero permitiendo evidenciar el trabajo científico y sistematizado en la asistencia. La favorable opinión de sus atributos por la mayoría de los participantes predice su aceptación en el ámbito formativo y asistencial.

## Resumo

**Introdução:**

A dificuldade na formulação de diagnósticos de enfermagem em estudantes e profissionais evidencia a necessidade de utilização de ferramentas baseadas em sistemas especialistas.

**Objetivo:**

Desenvolver um sistema especialista para formulação de diagnósticos de enfermagem e avaliar seus atributos.

**Materiais e métodos:**

Estudo tecnológico-descritivo, realizado em duas fases: projeto e construção do sistema especialista, e avaliação de seus atributos: usabilidade, funcionalidade, confiabilidade e portabilidade. Na segunda fase participaram 68 pessoas, entre estudantes e enfermeiros. Foram aplicados dois questionários, um para avaliação de usabilidade (validado por análise fatorial exploratória, com confiabilidade 0,93 Alfa de Cronbach) e outro para os demais atributos (validado com V de Aiken: 0,91; com confiabilidade: 0,91 Alfa de Cronbach). Os dados foram processados em Excel, aplicando-se estatística descritiva.

**Resultados:**

Foi desenhado um sistema especialista, utilizando a NANDA Internacional 2021-2023 como base de conhecimento. Sua interface permite inserir faixa etária, características e fatores, gerando etiquetas diagnósticas. A maioria dos usuários classificou os atributos usabilidade (79,41%), funcionalidade (82,35%), confiabilidade (77,94%) e portabilidade (86,76%) como muito bons.

**Discussão:**

O sistema especialista, DIEN versão 1, desenvolve habilidades para identificar características e fatores relacionados ou de risco, familiariza-se com a linguagem padronizada de enfermagem e fortalece o pensamento crítico para formular diagnósticos contextualizados para a pessoa cuidada.

**Conclusões:**

O DIEN versão 1 facilita a utilização de linguagem padronizada de diagnósticos de enfermagem, permitindo evidenciar o trabalho científico e sistematizado na assistência. A opinião favorável dos seus atributos pela maioria dos participantes prevê a sua aceitação na área da formação e da saúde.

## Introduction

 Nursing as a science and a profession applies an exclusive method in care known as the Nursing Process (NP)^1^, which ensures care based on a scientific background^2^. Five phases are interrelated, and nursing diagnosis is an essential pillar^3^. Nursing diagnosis is defined by the North American Nursing Diagnosis Association (NANDA) International^4^ as a “clinical judgment concerning a human response to health conditions/life processes, or a susceptibility to that response, that is recognized in an individual, caregiver, family, group or community” (p:193). 

 Various studies report that nursing diagnoses are not made explicit in the medical records (0.2%)2 or do not correspond to the interventions conducted^5^. Even when caring for people in critical condition, there is an 81% lower probability that the NP will be implemented^6^. It is also found that the diagnoses recorded in the medical records are not generated from the data obtained during the assessment, with psycho-emotional needs being barely assessed^7^. This situation hinders the comprehensive care of individuals and affects the visibility of the systematic and scientific work of nursing professionals.

 Insufficient nursing development in proposing diagnoses increases the risk of errors. This lack of development can be explained by multiple reasons, but the most prominent is the complexity to interrelate intellectual, technical, and personal skills^8^. At the undergraduate level, nurses are required to interpret human responses to vital processes or injuries. This indicates that students often experience difficulties in formulating NANDA-I diagnoses^9^ and use medical diagnoses, signs or symptoms as nursing diagnoses^10^.

 A different approach from training is required to overcome this situation. Such an approach should strengthen critical thinking and clinical reasoning, enabling the identification of defining characteristics, and their association with related or risk factors. This will allow the development of accurate nursing diagnoses, which are essential input for improving the quality of care and the competence of nursing professionals^11^-^13^.

 For more than four decades, many nursing schools have used NANDA-I to teach diagnosis. However, selecting the appropriate domain, class, and label remains a laborious process for students. Besides, analyzing the definition associated with its label, along with the defining characteristics and related factors is time-consuming and affects the timely and precise formulation of diagnoses, delaying the planning and execution of care interventions.

 In this respect, the nursing training process should include the use of computer tools. Students must use expert systems^14^,^15^ that contribute to the development of critical thinking and clinical reasoning, thereby strengthening their competence to diagnose human responses to health problems and vital processes. However, they have not been implemented in Peruvian universities yet, despite the advances in these systems and their application in health sciences^16^,^17^.

 Expert systems can transform the learning experience of nursing students and professionals. Therefore, the construction of an expert system to provide nursing diagnoses (DIEN Version 1) is a technological and educational contribution that will motivate users to utilize the NANDA International language and facilitate the development of competencies to formulate nursing diagnoses. Its implementation will make an impact in the quality of care, the training process and the nursing discipline. The objective of this study is to develop an expert system to formulate nursing diagnoses and evaluate its attributes.

## Material and Methods

**Design**

The design of this study consisted of an applied, descriptive technological piece of research developed in two phases: 1) the design and construction of the DIEN Expert System Version 1, and 2) the evaluation of the system attributes: usability, functionality, reliability, and portability, after training students and nurses^18^. 

For the construction of the system, the following steps were undertaken: a) domain and objectives definition, b) system architecture selection, c) knowledge base choice: NANDA-I 2021-2023 extracted using web scraping techniques, d) knowledge base design and implementation, e) web application development and inference engine, f) web platform integration, and g) tests to verify the attributes of the expert system implementation^19^. 

The evaluation of the expert system attributes was conducted with the participation of students and teaching/healthcare nurses. They participated in two online training sessions from January to February 2024. In these sessions, aspects related to the importance of nursing diagnosis, advantages, and use of the DIEN Version 1 were addressed. 

**Sample**

Convenience sampling was used. The students and teachers of the university where the study was conducted were invited through their institutional email, while healthcare nurses were invited through their personal email. The participants were 68 fourth- and fifth-year students enrolled in the second academic semester of 2023. Expert teachers in nursing diagnoses and healthcare nurses from Level II and III healthcare institutions in Lambayeque, Peru, were trained to use the DIEN Version 1 for evaluating these system attributes. 

**Instruments**

To assess the attributes of the DIEN Version 1, two Likert-type questionnaires were used. The first questionnaire evaluated usability with Cronbach's alpha reliability of 0.93 and validity determined by exploratory factor analysis using the maximum likelihood extraction method and the Oblimin rotation method. The second instrument evaluated functionality, reliability, and portability with a Cronbach's alpha reliability of 0.92 and global validity of 0.91 (Aiken's V). 

The questionnaire to assess usability consists of eleven items addressing interface and symbology aspects. The questionnaire assessing the remaining attributes is structured as follows: functionality, with five items addressing the essential components of nursing diagnoses (characteristics, factors, age group, diagnostic codes, and labels); reliability, with four items for identifying diagnostic labels (problem-focused, risk, health promotion, and syndrome); and portability, with two items about the ease of using the DIEN Version 1 on different electronic devices with internet access (laptops, PCs, or smartphones). 

Each item of the attribute is rated by the participant on a scale from 1 to 5, where 1 indicates “poor” and 5 “very good.” The assessment scores for each attribute are calculated by multiplying the number of items by the corresponding score (1 to 5), obtaining the limit of each category (very good, good, average and poor) according to the total score, as shown in Table 1. 

**Table 1:** General matrix of literature review

Assessment		Attributes		
	Usability	Functionality	Reliability	Portability
Deficient	11-12	5-9	4-7	2-3
Regular	22-32	10-14	8-11	4-5
Good	33-43	15-19	12-15	6-7
Very good	4-55	20-25	16-20	8-10

 Data collection for evaluating the expert system attributes was conducted online. Each participant was given a case study to solve using the DIEN Version 1 (https://dien.app). Subsequently, the participant was asked to evaluate the attributes of the expert system using specific questionnaires administered through Google Forms. Finally, the collected data were transferred to an Excel database for statistical processing.

**Data analysis **

 The variable “attributes of the DIEN Expert System Version 1 to formulate nursing diagnoses” was described in percentages using absolute and relative frequencies calculated in Excel. The collected data is freely accessible and available for consultation in the Harvard Dataverse, V1^20^.

**Ethical considerations**

 The participants' rights were protected through the informed consent process. They were invited via institutional or personal email to participate in the study, explaining the objective, the nature of their participation and the data collection technique. Those students who voluntarily agreed to participate in the research signed up for the training. The questionnaires were completed anonymously, and the data were only used for research purposes. The study was evaluated and approved by the Scientific Committee of the Research Unit of the Faculty of Nursing, then submitted to the Faculty Council, and ratified through Resolution No. 143-V -2022-CF-FE.

## Results

**Design and construction of the DIEN Expert System Version 1**

 The DIEN version 1 was completely developed following software engineering practices to ensure a robust and efficient system. The process included several critical steps:

- Domain and objectives definition: The system's objectives were initially defined. In this case, the field of nursing diagnoses was selected to develop an advanced technological solution to assist students and professionals in the diagnosis process.
- System architecture selection: Several architectures were considered for the expert system, including those based on rules, cases or frameworks. A relational database was chosen for the DIEN to store knowledge, while logic and interface were implemented using web components.
- Knowledge acquisition: The specialized knowledge of the NANDA-I taxonomy that classifies nursing diagnoses was gathered. Using web scraping techniques with a PHP script, information was extracted and structured from an online source for storage in a MySQL database. This process allowed the relationships between diagnoses, factors, and characteristics to be kept. The database registered 265 diagnostic labels with their respective numerical codes, 1286 related/risk factors, 1980 defining characteristics based on NANDA International 2021-2023, 2474 relationships between diagnoses and characteristics, and 2772 relationships between diagnoses and factors.

**Design and implementation of knowledge base**

PHP was used. This language is suitable for web platforms since it connects MySQL relational databases. 

**Web application and inference engine development**

 In this step, the user interface and web programming necessary to operate the expert system were developed, allowing interaction through a web browser. An essential component was the implementation of the inference engine, which analyzes the knowledge base to provide appropriate solutions or answers. The programming for this phase was done using PHP with the algorithm designed to process variables, such as age group (optional), relevant defining factors, and/or characteristics as inputs. To access the system, users must first log in to the system. Then, they can select the age group from a set of displayed options.

**Figure 1:**
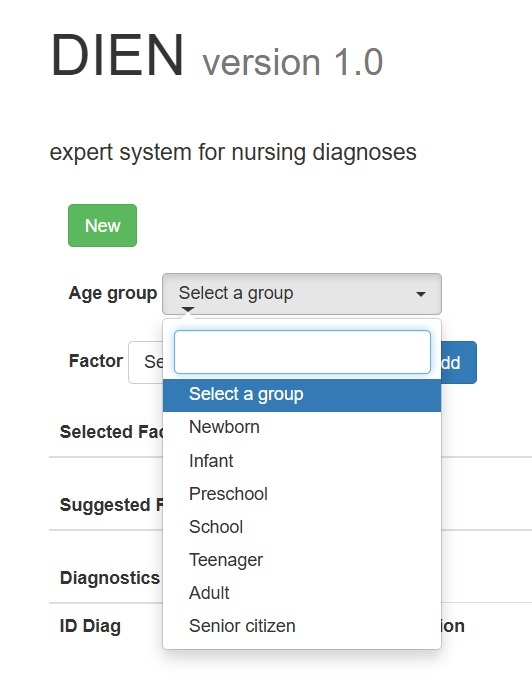
Age group drop-down list

**Figure 2:**
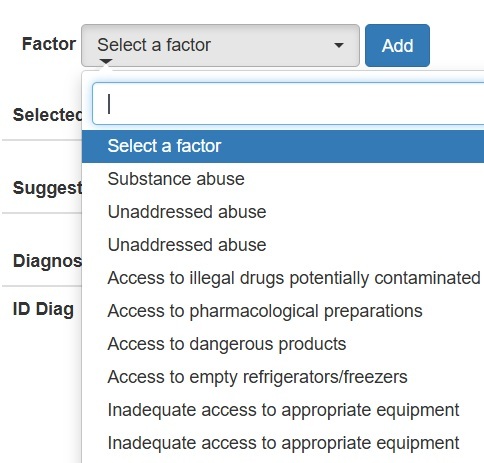
Example of a term in the “factor” list, displaying other factors based on the typed term

Next, using either the “Factor” or “Characteristic” list displayed on the interface, participants enter the corresponding data by clicking the "Add" button. They are able to type a key term or phrase to access a drop-down list containing a set of technical terms that correspond to the related or risk factors (Figures 2) used by NANDA-I, recording the selected data in the space.

**Figure 3:**
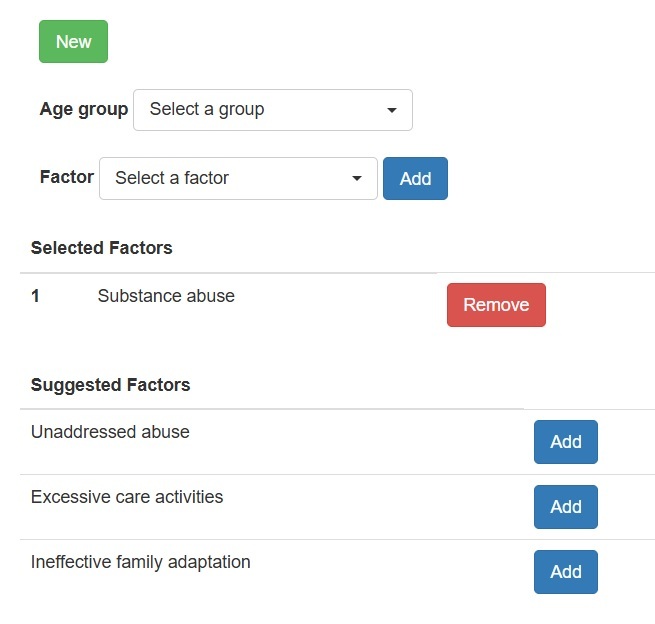
Suggested factors and buttons to add and remove a factor

 Users can delete a selected factor by clicking the “remove” button (Figures 3).

A similar procedure is followed with the list of “characteristics” that corresponds to the defining characteristics of the diagnoses. 

**Figure 4:**
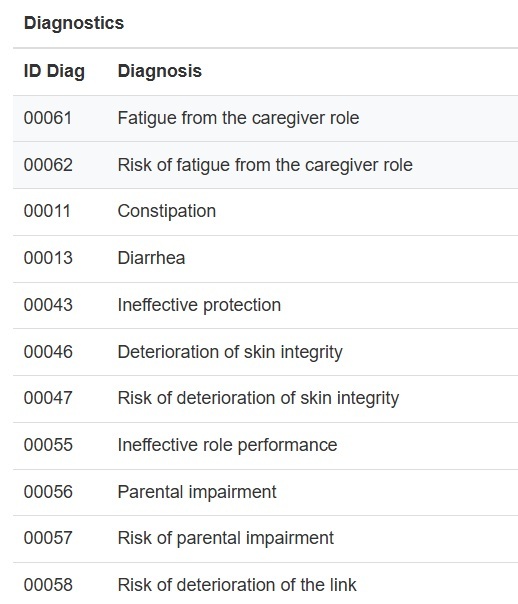
Diagnostic codes and labels

Based on the information provided by the user, the system searches for diagnostic labels that match the defining factors and characteristics entered by the user (Figures 4). Subsequently, those factors and characteristics not previously chosen are selected from these labels and offered as suggestions to the user. 

**Figure 5:**
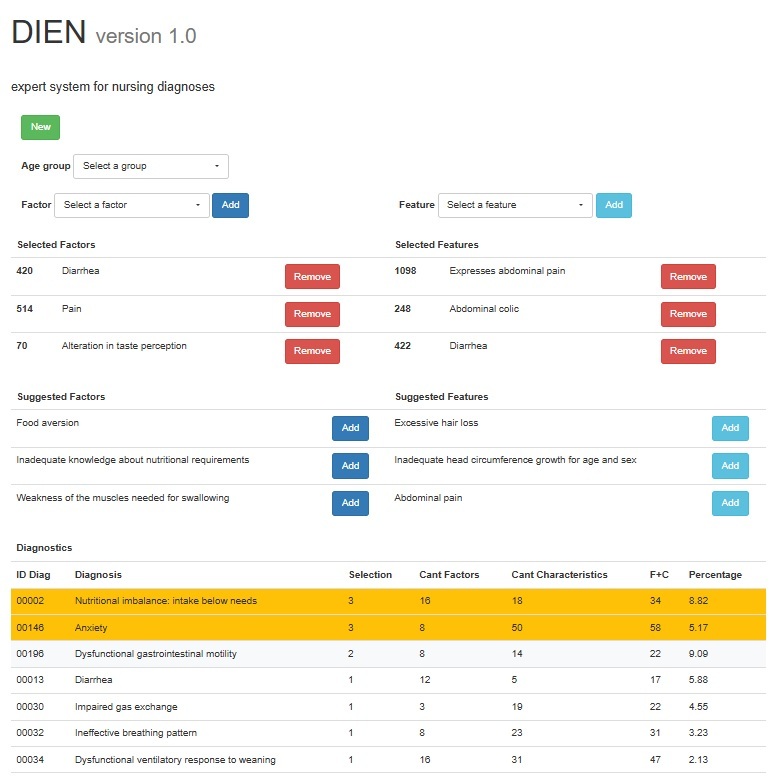
The DIEN Expert System Version 1 user interface

Additionally, the inference engine performs the necessary statistical calculations to generate a list of suggested diagnoses, thus optimizing the assistance provided to nursing students and professionals in formulating accurate and substantiated diagnoses (Figures 5). 

The user who performs a critical analysis has the possibility of choosing the diagnostic labels with their respective codes contextualized to the case under study, allowing them to identify from the label headings the diagnoses focused on the problem (which specifies the human response), those of risk (which precedes the phrase Risk for...), those of health promotion (which precedes the phrase willingness to improve...) or those of syndrome (which precedes the phrase syndrome of...). 

With this information, users can quickly identify the domain and class to which the diagnostic label corresponds. They can then analyze the definition of the diagnosis and connect it with the appropriate defining characteristics, related factors, or risk factors.

**Testing and validation**

The testing process included unit, integration, system, and usability checks. Frameworks such as PHP Unit were used for unit testing, ensuring the correct interaction between components and the general functionality of the system in a production-like environment. Usability tests allowed us to evaluate the interface and symbology criteria. Subsequently, functionality, reliability, and portability attributes were evaluated. 

**Implementation and maintenance**

Finally, the system was deployed on a virtual private server (VPS), configuring a web server and a MySQL database manager to facilitate the implementation and operation of the DIEN Expert System. 

This free tool is available to nursing students and professionals and can be accessed through the link: https://dien.app/. The screen has an interface and symbols that make it very user-friendly. 

**System attributes evaluation: usability, functionality, reliability and portability after training students and nurses**

Most users rated the qualities of DIEN Version 1 as “very good”: usability (79.41%), functionality (82.35%), reliability (77.94%) and portability (86.76%), as shown in the following Table 2. 

**Table 2:** Patterns associated with domestic violence

Assessment		Attributes		
	Usability	Functionality	Reliability	Portability
Deficient	2.94	2.94	2.94	1.47
Regular	1.47	1.47	2.94	4.41
Good	16.18	13.24	16.18	7.35
Very good	79.41	82.35	77.94	86.76

## Discussion

The incorporation of ICT in people's health care represents an invaluable resource to improve quality of life at any life cycle stage considering the different care needs^21^-^23^. In this context, the use of Artificial Intelligence (AI) stands out, as it allows the development of prototypes for personal care, optimizes decision-making and service management, and improves the quality of care. In addition, AI encourages critical and reflective thinking, as well as professional confidence^24^. 

On the one hand, these advances require training institutions to renew educational processes by integrating ICT, research, and clinical practice^25^-^27^ to meet social expectations for competent professionals capable of making quick and accurate decisions in complex situations. On the other hand, students prefer to access information through technological tools due to the fact that they are digital natives. They have adopted e-learning strategies and the use of mobile applications and clinical simulators, which are essential tools to improve educational quality^28^. When combined with expert systems, these tools open new horizons in the teaching and learning of the NP, strengthening key competencies of the nursing professionals^29^,^30^. 

Within this framework, the DIEN Expert System Version 1 was designed based on NANDA-I, the language most commonly used in nursing practice. The system applies knowledge and reasoning techniques to solve problems that can normally be solved by experts of the field^31^. When it comes to nursing diagnoses, the expert is NANDA-I, a language that meets the criteria established by the Committee for Nursing Practice Information Infrastructure (CNPII) of the American Nurses Association (ANA)^32^. 

As recommended by UNESCO^33^, the DIEN Version 1 will be updated according to future editions of the NANDA Taxonomy I and will strengthen the student's cognitive abilities. It will develop skills to identify defining characteristics, related factors, and risk factors^34^. In addition, it will provide the opportunity to expand basic knowledge of NANDA-I technical language, reinforce critical and reflective thinking^35^, and strengthen clinical judgment. This, in turn, will contribute to the formulation of precise nursing diagnoses and facilitate the continuity of care, making visible the autonomous and scientific work of the nursing profession. 

Although limited to specific cases such as schizophrenia, Sánchez Hernández's study^36^ showed the effectiveness of expert systems based on NANDA-I, NOC, and NIC. In contrast, the DIEN Version 1 has a broader scope, allowing its application to any patient, scenario, or life cycle stage. Furthermore, it is free and universally accessible, overcoming the limitations of other systems, such as software restricted to specific institutions. 

Hernández-Herrera et al.^37^ developed software for the application of the NP during its assessment and diagnosis phases for undergraduates, but access was limited to students from the institution, and it was not evaluated by the users. In contrast, the DIEN Version 1 offers universal and free access and its attributes has been evaluated by end users as “very good”. 

Its usability was rated considering its efficient and intuitive design^38^,^39^. In terms of functionality, participants considered that the lists and buttons enabled them to enter data on the age group, related factors or risk factors, and defining characteristics. This process generates a set of diagnostic labels that could be selected based on critical judgment and the contextualization of the person being cared for, facilitating the formulation of nursing diagnoses. This system was also tested using the black box testing^40^ in which participants gave satisfactory results after its application. 

The system's reliability was also evaluated as “very good.” The participants noted the diagnostic codes and labels, as well as the different types of nursing diagnoses to be identified. This precision facilitates the location of the domain and class within NANDA-I, followed by the analysis of the characteristics alongside related and risk factors. This process leads to the identification of different types of nursing diagnoses:

- *Problem-focused diagnosis:* When related factors and defining characteristics are added to the label that reveals an undesirable human response.
- *Risk diagnosis:* When a label that begins with “risk for” is followed by the undesirable human response related to the factor(s) that increase the person's vulnerability.
- *Health promotion diagnosis:* When the human response and defining characteristics reflecting a desire to improve a behavior are added to a label beginning with “willingness to improve.”
- *Syndrome diagnosis:* When the label begins with “syndrome of” and is related to the set of nursing diagnoses that occur and are addressed through similar interventions.

Portability was confirmed by participants who verified that this system can be used on multiple devices: personal computers, laptops, tablets, or cell phones, without substantial changes. Designed using HTML, JavaScript, and cascading style sheets (CSS), the system adheres to web standards.

 The study by Aristoteles et al.^31^, which developed an expert system for nursing students to learn NP based on an Android platform and was positively assessed by most participants (84%), even though it did not have the characteristics of the DIEN Version 1. This result indicates that such tools are well accepted by the academic community, as they facilitate the processing of large volumes of data and support decision-making that contributes to the humanization of care^41^,^42^.

 Therefore, the DIEN Version 1 becomes indispensable for professional training, not only at the undergraduate level but also in second specialties and at the postgraduate studies. This tool, registered with the National Institute for the Defense of Competition and the Protection of Intellectual Property (INDECOPI) of Peru, is available to students and nursing professionals as an app. However, it requires the commitment and training of students and teachers, digital natives and digital immigrants, respectively^43^ for its understanding, adaptation, and immediate use. 

 The scarce publications on this topic invites us to conduct other studies that relate to the nursing students’ cognitive processes, which, together with the empathy and human understanding that characterizes nursing professionals, will ensure the humanization of care. With the advances in AI and expert systems, it is recommended that future studies incorporate new techniques, such as large language models (LLM) to optimize user-system interaction and streamline decision-making processes.

 We point out the following limitations of the study. First, the multiple translations of NANDA-I required numerous search engine revisions. Secondly, the time allocated for training in the use of the system has been insufficient for participants to familiarize themselves fully with this tool. Thirdly, teachers' limited experience with nursing diagnoses and the application of AI restricted the integration of the DIEN Version 1 into training processes. Fourthly, reluctance to use nursing diagnoses in healthcare practice does not allow us to assess the importance of this system. Finally, the language in which the expert system has been designed limits its use by students and nurses who do not speak Spanish.

## Conclusions

The attributes usability, functionality, reliability and portability of the DIEN Expert System Version 1 were positively rated by most participants. Incorporating this expert system into the training of nurses will facilitate the development of competencies for formulating nursing diagnoses using the standardized NANDA-I language. In addition, it will strengthen critical thinking, decision-making, and the contextualization of care. 

The positive evaluation of this expert system by the participants predicts its wide acceptance, as it is perceived as a tool that will revolutionize the teaching and learning of nursing diagnoses. Finally, the DIEN Version 1 can also be used in the field of care to facilitate precision in nursing diagnoses and continuity of care. This will contribute to making visible the nursing professional's scientific work. 
